# Comprehensive expressional analyses of antisense transcripts in colon cancer tissues using artificial antisense probes

**DOI:** 10.1186/1755-8794-4-42

**Published:** 2011-05-16

**Authors:** Rintaro Saito, Keisuke Kohno, Yuki Okada, Yuko Osada, Koji Numata, Chihiro Kohama, Kazufumi Watanabe, Hajime Nakaoka, Naoyuki Yamamoto, Akio Kanai, Hiroshi Yasue, Soichiro Murata, Kuniya Abe, Masaru Tomita, Nobuhiro Ohkohchi, Hidenori Kiyosawa

**Affiliations:** 1 Institute for Advanced Biosciences, Keio University, Tsuruoka 997-0017, Japan; 2 Department of Surgery, Graduate School of Comprehensive Human Sciences, University of Tsukuba, Ibaraki 305-8575, Japan; 3 Systems Biology Program, Graduate School of Media and Governance, Keio University, Fujisawa 252-8520, Japan; 4 Technology and Development Team for Mammalian Cellular Dynamics, BioResource Center (BRC), RIKEN Tsukuba Institute, Ibaraki 305-0074, Japan; 5 Graduate School of Life and Environmental Sciences, University of Tsukuba, Ibaraki 305-0006, Japan; 6 Custom Biotechnology Service Group, Hokkaido System Science Co. Ltd, 2-1 Shinkawa Nishi 2-1, Kita-ku, Sapporo 001-0932, Japan; 7 C's NEXT Co. Ltd. Maruito Kita 4-jyou Bldg., 4F, Kita 4-jyou Higashi 2-8-2, Tyuo-ku, Sapporo 060-0034, Japan; 8 Faculty of Environment and Information Studies, Keio University, Fujisawa, 252-8520, Japan; 9 National Institute of Agrobiological Sciences, Tsukuba, 305-8602, Japan; 10 Inquiries regarding clinical aspects of the samples should be addressed to N.O; 11 Laboratory of Cellular Biochemistry, Department of Animal Resource Sciences, Graduate School of Agricultural and Life Sciences The University of Tokyo, Yayoi 1-1-1, Bunkyo-ku, Tokyo, 113-8657, Japan

## Abstract

**Background:**

Recent studies have identified thousands of sense-antisense gene pairs across different genomes by computational mapping of cDNA sequences. These studies have shown that approximately 25% of all transcriptional units in the human and mouse genomes are involved in *cis*-sense-antisense pairs. However, the number of known sense-antisense pairs remains limited because currently available cDNA sequences represent only a fraction of the total number of transcripts comprising the transcriptome of each cell type.

**Methods:**

To discover novel antisense transcripts encoded in the antisense strand of important genes, such as cancer-related genes, we conducted expression analyses of antisense transcripts using our custom microarray platform along with 2376 probes designed specifically to detect the potential antisense transcripts of 501 well-known genes suitable for cancer research.

**Results:**

Using colon cancer tissue and normal tissue surrounding the cancer tissue obtained from 6 patients, we found that antisense transcripts without poly(A) tails are expressed from approximately 80% of these well-known genes. This observation is consistent with our previous finding that many antisense transcripts expressed in a cell are poly(A)-. We also identified 101 and 71 antisense probes displaying a high level of expression specifically in normal and cancer tissues respectively.

**Conclusion:**

Our microarray analysis identified novel antisense transcripts with expression profiles specific to cancer tissue, some of which might play a role in the regulatory networks underlying oncogenesis and thus are potential targets for further experimental validation. Our microarray data are available at http://www.brc.riken.go.jp/ncrna2007/viewer-Saito-01/index.html.

## Background

Non-coding RNAs are one class of RNAs that do not encode proteins but have specific cellular activities. Some non-coding RNAs are antisense RNAs encoded on the antisense strand of protein-coding genes. Recent progress in sequencing technologies has allowed the rapid sequence analysis of the large amount of RNAs that are transcribed in the cell. For example, the large-scale cDNA sequencing projects conducted by the FANTOM consortium revealed that a large proportion of the mouse genome is transcribed into RNAs and that many of these RNAs do not have protein-coding potential and thus are considered non-coding RNAs [1,2]. Studies using tiling arrays further support these observations and have revealed the presence of RNA-encoding regions in the genome by computational mapping [3]. Computational mapping has revealed that many RNAs are located on the antisense strand of the protein-coding genes in the same genomic region; approximately 25% of transcriptional units are involved in *cis*-sense-antisense gene pairs in the human and mouse genomes, thus suggesting the presence of more sense-antisense gene pairs than previously thought [4-9]. Although the experimentally determined functions of antisense RNAs are limited, some have been shown to regulate transcription from the sense strand [6]. Thus, expressional analyses of sense-antisense transcripts may shed light on the mechanisms underlying the control of these transcripts.

In our previous studies [7,10], we conducted comprehensive expressional analyses of sense-antisense transcripts in human and mouse cells. In these studies large amounts of transcripts were detected by the random priming method, but not by the oligo-dT priming method, thus suggesting that these transcripts are poly(A)- and not reflected by cDNA sequences, which rely on the existence of poly(A) tails. Comprehensive cluster analysis of the expression ratio of sense and antisense transcripts is a powerful approach to characterize expression patterns of sense-antisense transcripts in various tissues. We found that expression balances of some of these transcripts were altered in specific tissues. Furthermore, Northern hybridization analysis of several selected sense-antisense transcripts showed smeary hybridization patterns in the mouse, thus indicating that the transcribed region in the genome may vary and that the cDNA sequences do not always reflect the actual sizes of the transcripts. Therefore, the design of microarray probes based on cDNA sequences obtained by large-scale cDNA sequencing projects is not appropriate for the detection of all transcripts comprising the transcriptome.

Subsequently, we designed microarray probes referred to as artificial antisense sequence (AFAS) probes specific for sequences in the antisense strand of known transcription units originating from the sense strand. In this way, we were able to detect the expression of novel antisense RNAs, which were not detected by using cDNA sequences [11]. These AFAS probes were spotted onto microarray slides and used in our microarray platform to generate expression data for the analysis of antisense transcripts. Using mouse adult tissues, we previously found that 66.1% of 635 genes targeted for such expressional analysis showed positive expression from the antisense strand, suggesting that our AFAS probes can efficiently detect novel antisense transcripts [11].

We then chose mouse cancer tissues for expressional analyses of sense-antisense transcripts, because most oncogenesis is caused by abnormalities in gene sequences or in the regulatory systems controlling gene expression, and possibly also by unknown RNAs transcribed from the antisense strand. Uncovering how gene expression is regulated in cancer cells may lead us to a better understanding of the mechanisms and signaling networks underlying oncogenesis and metastasis [12]. Several early studies succeeded in identifying genes expressed specifically in cancer cells; however, most studies have focused on investigating the expression of messenger RNAs, which encode proteins [13-17].

Several studies have identified non-coding RNAs expressed specifically in cancer cells [18,19]; however, those focused on antisense RNAs are limited [20,21]. Using AFAS probes, we found that expression of antisense transcripts from 95 well-annotated genes showed altered expression level in cancer tissues [11]. Some of their expressions were validated using RT-PCR, Northern blot analysis, and *in situ *hybridization, thus suggesting that our platform can efficiently detect novel antisense transcripts, some of which may be regulated in cancer cells.

Here, using the same microarray platform, we analyzed expression patterns of antisense transcripts in human tissue. First we characterized the expression patterns of sense-antisense transcripts, based on available cDNA sequences, in colon (colorectal) cancer tissues and in normal tissues surrounding the cancer tissues. Although expression balances (ratios) of most of sense and antisense transcript pairs did not change between patients or between normal and cancer tissues, we found 68 sense-antisense transcripts whose expression balances were altered specifically in colon cancer tissues. Then, to identify novel antisense RNAs expressed in cancer tissues, we used the AFAS probes to analyze the expression patterns of sequences in the antisense strand of well-known protein-coding genes that are suitable for cancer research as defined in the Atlas Human Cancer 1.2 Array. Sixty-mer probes were designed for every 500 bps of the antisense strand of protein-coding genes. We used the oligo-dT and random priming methods for transcript generation in order to screen both the poly(A)+ and poly(A)- transcript populations in total RNA extracted from colon cancer samples obtained from 6 patients.

Using random priming, we detected gene expression with approximately 40% of the AFAS probes, which correspond to approximately 80% of the well-known genes. Among them, 172 probes detected twofold differences in expression levels of antisense transcripts between cancer tissue and normal tissue, demonstrating the usefulness of our approach to discover novel antisense transcripts, some of which might be related to the regulation of gene expression in cancer cells. Although we used only colon cancer tissues in this study, we believe that our approach is applicable to other types of cancer tissues.

## Results

### The expression balance of 68 cDNA-based sense-antisense transcripts is altered in colon cancer tissues

We previously established a custom microarray platform (designated as 11 k) to analyze the expression patterns of sense-antisense transcripts in human and mouse [10,11]. These sense-antisense transcripts were identified on the basis of available cDNA sequences as [10]. For validation of this microarray platform, three sense-antisense pairs were selected for Northern and *in situ *hybridization analyses to confirm their transcription [7,10]. Here, we conducted DNA microarray analyses by using the same set of probes designed for 2621 sense-antisense pairs in [10] to detect transcripts expressed in colon cancer tissues. These probes comprise 2358 pairs for the detection of protein-coding transcripts only, 250 pairs for the detection of protein-coding transcripts paired with non-protein-coding transcripts, and 13 pairs for the detection of non-protein-coding transcripts only. Protein-coding transcripts, as defined in our previous study [10], are those giving a significant hit to known protein sequences by BLASTX searches (homology search from DNA to protein). Otherwise, we defined the transcripts as non-coding. Total RNAs were extracted from colon cancer tissue and normal tissue surrounding the cancer tissue obtained from 6 patients. Fluorescence labeling of transcripts was performed by using both oligo-dT and random priming methods.

**Figure 1:**
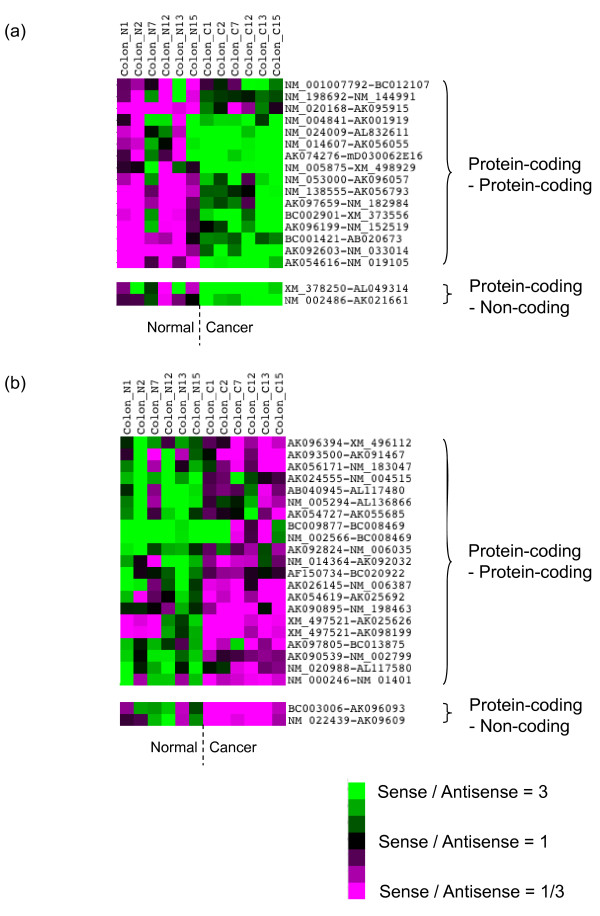
**Clustering analysis of the expression ratio of the sense and antisense pair of transcripts showing altered expression balances**. Only the clustering result obtained by the random priming approach is shown. The green color indicates higher expression (threefold or more) of the sense transcript compared with its antisense counterpart. The red color indicates lower expression of the sense transcript (1/3-fold or less) compared with its antisense counterpart. (a) Clustering result for sense-antisense pairs where the sense gene is up-regulated and the corresponding antisense gene is down-regulated in cancer tissues. (b) Clustering result for sense-antisense pairs where the sense gene is down-regulated and the corresponding antisense gene is up-regulated in cancer tissues.

Microarray data analysis revealed that the expression ratios between sense and antisense transcripts were clustered, as shown in our previous studies (Additional file 1: Figure S1) [7,10]. Although most of the expression ratios between sense and antisense transcripts did not change between patients or between normal and cancer tissues, we found sense-antisense transcripts showing altered expression balances in colon cancer tissues. For example, Figure 1 (also listed in Additional file 2: Table S1) shows that the expression balances of 68 sense-antisense transcript pairs were reversed in cancer tissues. For 29 pairs among them, the sense transcripts showed a >10% increase in expression levels in cancer tissues, whereas the corresponding antisense transcripts showed a similar decrease in at least three of six patients (according to expression data obtained by random priming). For the remaining 39 pairs, the expression levels of the sense transcripts was >10% greater in normal tissues than in cancer tissues, whereas the corresponding antisense transcripts showed a similar level of increased expression in cancer tissues. We analyzed functional annotations (GO, Gene Ontology [22]) of genes corresponding to these sense-antisense pairs. The cellular processes in which these genes are involved were biased toward signal transduction, cell adhesion, and cell differentiation, which play important roles in oncogenesis and metastasis (Additional file 2: Table S2). The up-regulation and down-regulation of sense-antisense transcripts can be more precisely assessed by analysis of the expression ratios between sense and antisense transcripts in normal and cancer tissues (Additional file 1: Figure S2).

In this analysis, we identified six sense-antisense gene pairs in which the sense gene was protein-coding and the antisense gene was non-coding, as defined previously [10]. Interestingly, two of the sense genes were involved in cell differentiation, which may be related to cancer. Subsequently, we screened for novel non-coding transcripts that might be encoded by the antisense strand. Our previous work showed that vast amounts of transcripts were poly(A)- [7,10] and thus they may not be detected by a cDNA-based approach. Therefore, we screened for these novel transcripts by using the AFAS probes, which could detect poly(A)- transcripts by using random priming approach.

### AFAS transcripts are expressed from about 80% of the well-known genes and most are poly(A)-

**Figure 2:**
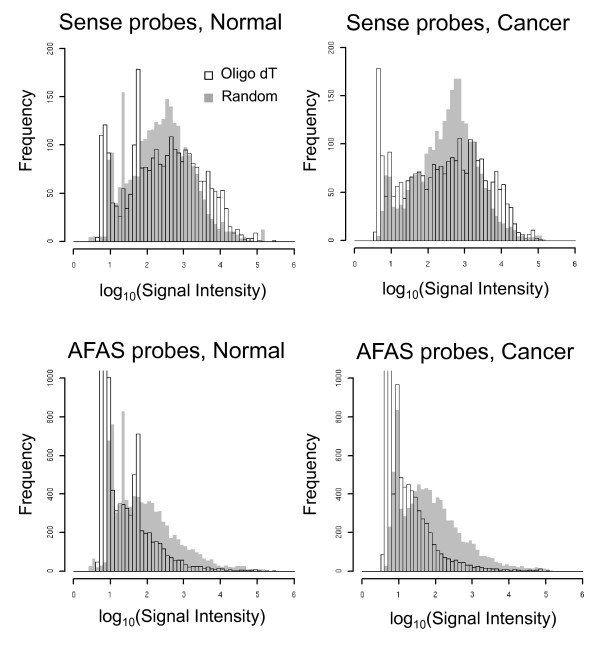
**Distributions of microarray signal intensities obtained using sense (top) and AFAS probes (bottom)**. Signals obtained by the oligo-dT priming or by the random priming approach are indicated. The graphs on the left represent the expression distributions in normal tissues, whereas those on the right represent the expression distributions in cancer tissues.

Our established microarray platform (custom microarray platform 11 k) comprises 501 probes covering well-known genes suitable for cancer research, including oncogenes and tumor suppressors. We obtained expression data for cancer tissue obtained from 6 patients and validated these data by comparison with previously obtained data, as described in the methods section. Our microarray platform also contains 2376 AFAS probes designed to correspond to every 500 bases of the antisense strand of exonic regions of well-known genes suitable for cancer research to screen for transcripts originating from the antisense strand. These genomic regions, genes encoded in these regions, and transcripts originating from these regions, were designated as AFAS regions, AFAS genes, and AFAS transcripts, respectively. The AFAS probes detected fewer transcripts than the probes corresponding to protein coding genes on the sense strand. Although most of the AFAS regions were not transcribed, or were expressed only at low levels, the detection of transcripts by the AFAS probes implies the existence of antisense genes (Figure 2). For example, for normal and cancer tissue, 14.8% and 8.3% of AFAS probes, respectively, detected expression intensities satisfying a conservative threshold (≥100)[11], according to expression data obtained by oligo-dT priming. As expected, the sense probes detected markedly more transcripts, with 64.1% and 64.2% of the sense probes detecting expression intensities in normal and cancer tissues, respectively, that satisfy the same threshold. By comparison, more transcripts were detected with AFAS probes by using the random priming approach to prepare target cDNAs; approximately 39.4% and 38.2% of the AFAS probes satisfied the conservative threshold (≥100) in normal and cancer tissues, respectively, by using random priming (*P *= 7.19×10^-1061^, χ^2 ^test). The rates of probes detecting positive expression according to more moderate criteria provided by Agilent's platform are summarized in Additional file 2: Table S3. Figure 2 shows the higher overall expression levels detected by AFAS probes using random priming compared with oligo dT priming in both normal and cancer tissues. Since oligo-dT priming is expected to label mRNAs, including unspliced pre-mRNAs having poly(A) tails, whereas random priming is expected to label all RNAs in a cell, these results indicate that most of the transcripts originating from the AFAS regions (AFAS transcripts) are poly(A)-. This finding is consistent with the results of a previous study showing that the majority of sense-antisense transcripts are poly(A)- [7]. The possibility that these poly(A)- transcripts are localized in the nucleus must be confirmed by biological experiments. It is noteworthy that publicly available cDNA sequences originating from the antisense strand can be found for only 139 (27.7%) of the 501 well-known genes used in the present study. This calculation is based on the set of cDNA sequences used in our previous work [11]. Approximately 70% of the AFAS probes that detected gene expression did not correspond to these cDNA sequences. Thus, we suggest that these probes correspond to potentially novel antisense RNAs. At least one AFAS probe showed a median expression level (≥100), using random priming, for 78.2% and 77.4% of the well-known genes in normal and cancer tissues, respectively. Although cross-hybridization of AFAS probes with transcripts must be considered, our results reveal that our microarray platform utilizing the AFAS probes and the random priming method to prepare transcripts has the capacity to detect novel transcripts from the antisense strand. Therefore, for subsequent analyses, we focused on the expression data using random priming to investigate the possible regulatory functions of identified poly(A)- RNAs.

### The expressional level of 172 transcripts identified with the AFAS probes is altered in colon cancer tissue

Transcripts identified by AFAS probes with altered expression levels in cancer tissue might play a role in oncogenesis. Therefore, we first calculated the ratio of the median expressional level between normal tissues and cancer tissues (cancer to normal ratio) for each sense and AFAS transcript (Additional file 1: Figure S3). We found many putative antisense genes (AFAS genes, detected by the AFAS probes) with altered expression in cancer tissues compared with normal tissue. We identified 71 AFAS transcripts expressed more than twofold higher in colon cancer tissues than in normal tissues and 101 AFAS transcripts expressed in colon cancer tissues at a level less than 50% of the level found in normal tissue. These numbers were much greater than the expected number of transcripts, which was identified as 50.14 ± 6.13 S.D by the random shuffling test.

**Figure 3:**
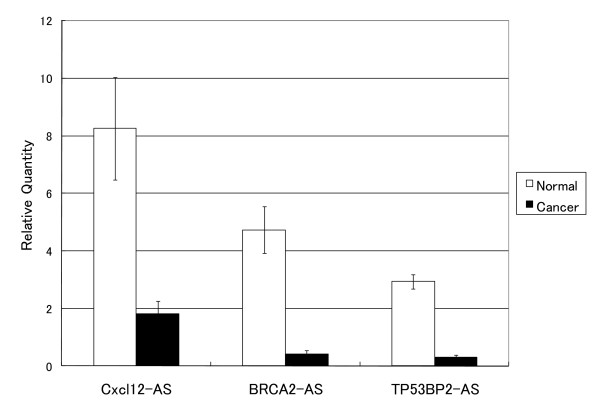
**Expression levels of three AFAS transcripts in normal and cancer tissues measured by RT-PCR**. Sense gene symbols followed by "-AS" are shown on the horizontal axis. The error bars reflect the standard deviation of the technical duplicates.

We chose three AFAS probes that showed alterations in expression level in cancer tissue by microarray analysis and conducted RT-PCR to verify these results. Our RT-PCR results (Figure 3) showed that for all three antisense probes there were notable changes in expression level between cancer tissues and normal tissues.

We also analyzed how the expression level of sense transcript changes in response to the expressional changes of antisense transcripts detected by AFAS probes. When the oligo-dT priming method was used to prepare the transcripts, the number of sense-AFAS pairs that showed a concerted increase or decrease in expression level in cancer and normal tissues was 1451, which is statistically higher than the expected number of 1260.19 (*P *= 2.0×10^-20^, χ^2 ^test). This finding is consistent with the results of a previous study showing a positive correlation between the expression of sense and antisense transcripts [4,20]. However, no such positive correlation was observed for expression data obtained by random priming.

The cancer to normal ratio for the sense and antisense transcript in each pair can be used to detect sense-antisense transcript pairs having correlated or anti-correlated expression, as shown in Additional file 1: Figure S3 for random priming. For example, there are 21 sense-AFAS pairs where expression of the sense transcript is increased twofold or more while the expression of the AFAS transcript is decreased by 50% or more in cancer tissues (plots in the lower right of the fourth quadrant of Additional file 1: Figure S3).

The sense to antisense ratio is a powerful measure to characterize the expression balance of the sense and antisense genes in particular genomic regions as demonstrated by our previous studies [7,10]. To obtain a global picture of changes in such expression balances for the sense-AFAS pairs, we conducted clustering analyses. In particular, for data obtained by random priming we calculated the sense to AFAS expression ratio for each sense-AFAS pair and conducted hierarchical clustering (Additional file 1: Figure S4). By aligning the clustering results, we then compared the expression balance patterns for data obtained by oligo-dT priming with those obtained by random priming for each sense-antisense pair.

Most of the AFAS probes revealed lower expression levels than did the sense probes in both cancer tissue and normal tissue, especially for the expression data obtained by oligo-dT priming. However, by using the random priming approach we detected 306 sense-AFAS pairs for which the AFAS probes detected expression levels three times (according to the median expression level among the patients) those detected by the corresponding sense probes, in both cancer tissue and normal tissue. In contrast, only 91 sense-AFAS pairs were detected by the same criteria using oligo-dT priming (*P *= 1.81×10^-29^, χ^2 ^test). This observation mostly corresponds to the red region in the upper part of the heat map designated as "random" in Additional file 1: Figure S4.

**Figure 4:**
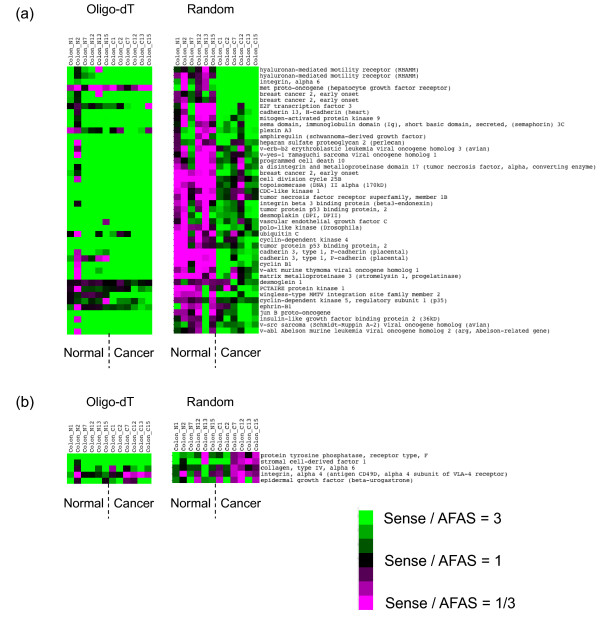
**Clustering analysis of the expression ratio of well-known genes (sense transcripts) and their corresponding AFAS transcripts showing altered expression balances**. Clustering analysis was conducted for expression ratios obtained by the random priming approach. Expression ratios obtained by the oligo-dT priming approach were aligned to the clustering results of the random priming approach. The green color indicates higher expression detected by the sense probe compared with the corresponding AFAS probe (3-fold of more). The red color indicates lower expression detected by the sense probe compared with its antisense counterpart (1/3-fold of less). Sense-AFAS probe pairs in the figure correspond to those listed in Table 1. (a) Clustering result for sense-AFAS pairs where the sense gene is up-regulated and the corresponding AFAS gene is down-regulated in cancer tissues. (b) Clustering result for sense-AFAS pairs where sense gene is down-regulated and the corresponding antisense gene is up-regulated in cancer tissues.

We then observed sense-AFAS probe pairs having altered expression balances among normal and cancer tissues; for example there were 44 sense-AFAS probe pairs for which the sense transcript showed higher expression than AFAS transcript in cancer tissues but the balance was reversed in normal tissues for at least three of the six patients (Figure 4a and Table 1). We noted that for these 44 sense-AFAS pairs, there were at least 10% differences in expression levels detected by sense and AFAS probes, and both sense and AFAS probes showed at least a 10% change in expression level between normal and cancer tissues, for these three patients. The observed number, 44, was much higher than the expected number 12.07 ± 3.11 SD (Random shuffling test). If the threshold of 10% was changed to 20% or 30%, the observed number of pairs decreased to 35 and 21, respectively, although the rate of biologically relevant pairs within these pairs might increase. We also screened for sense-AFAS probe pairs for which the sense transcript showed lower expression than the AFAS transcripts in cancer tissues but for which the balance was reversed in normal tissues. We obtained five pairs consistent with this scenario (Figure 4b and Table 1). We also identified sense-AFAS transcript pairs for which the sense transcript showed higher (lower) expression than that of the AFAS gene, in cancer tissues, where expression levels were based on expression data from oligo-dT priming for sense transcripts and random priming for AFAS transcripts (Additional file 2: Table S4ab). This procedure might be rationalized by the better detection of protein-coding gene expression by using the oligo-dT priming approach and, conversely, the better detection of RNA expression by the random priming approach. Although transcripts detected by oligo-dT priming and those detected by random priming methods may function in different cellular processes, they might be associated at the level of gene expression. The sense-AFAS probe pairs listed in Table 1 are also indicated in Additional file 1: Figure S3 (designated as Sense up, AFAS down, corresponding to the first 44 pairs in Table 1 and Sense down, AFAS up, corresponding to the last five pairs in Table 1).

**Table 1:** List of sense-AFAS probe pairs detecting altered expression balances in normal tissue and cancer tissue in patients with colon cancer based on the random priming approach

AFAS ID	Diff. (%)	Annotation
X07876-03	97.2	wingless-type MMTV integration site family member 2
L31951-03	94.1	mitogen-activated protein kinase 9
U29343-04	74.6	hyaluronan-mediated motility receptor (RHAMM)
U09304-01	63.3	ephrin-B1
U43746-02	62.6	breast cancer 2, early onset
X63629-03	57.8	cadherin 3, type 1, P-cadherin (placental)
M25753-01	54.5	cyclin B1
U69611-04	50.7	a disintegrin and metalloproteinase domain 17 (tumor necrosis factor, alpha, converting enzyme)
M63167-01	47.9	v-akt murine thymoma viral oncogene homolog 1
M77830-02	43.9	desmoplakin (DPI, DPII)
M29366-04	40.9	v-erb-b2 erythroblastic leukemia viral oncogene homolog 3 (avian)
M15990-01	39.3	v-yes-1 Yamaguchi sarcoma viral oncogene homolog 1
X53586-04	38.4	integrin, alpha 6
U43746-15	37.1	breast cancer 2, early onset
J04088-07	36.5	topoisomerase (DNA) II alpha (170 kD)
X87852-02	36.5	plexin A3
X05232-03	32.7	matrix metalloproteinase 3 (stromelysin 1, progelatinase)
M29039-02	32.7	jun B proto-oncogene
X80343-01	32.4	cyclin-dependent kinase 5, regulatory subunit 1 (p35)
L34058-01	30.3	cadherin 13, H-cadherin (heart)
Y10479-01	30.3	E2F transcription factor 3
M14505-02	29.7	cyclin-dependent kinase 4
J02958-06	29.7	met proto-oncogene (hepatocyte growth factor receptor)
U43746-11	29.1	breast cancer 2, early onset
U58334-01	29.1	tumor protein p53 binding protein, 2
X63629-01	28.5	cadherin 3, type 1, P-cadherin (placental)
U58334-02	28.2	tumor protein p53 binding protein, 2
X56654-01	27.9	desmoglein 1
M81934-01	27.6	cell division cycle 25B
X66363-03	27.6	PCTAIRE protein kinase 1
M26880-04	26.8	ubiquitin C
U37139-02	25.6	integrin beta 3 binding protein (beta3-endonexin)
L29222-02	24.2	CDC-like kinase 1
U01038-02	21.9	polo-like kinase (Drosophila)
M35410-02	21.1	insulin-like growth factor binding protein 2 (36 kD)
M35296-07	18.9	v-abl Abelson murine leukemia viral oncogene homolog 2 (arg, Abelson-related gene)
AF022385-02	18.6	programmed cell death 10
M30704-01	17.5	amphiregulin (schwannoma-derived growth factor)
U43142-01	16.1	vascular endothelial growth factor C
M32315-05	15.9	tumor necrosis factor receptor superfamily, member 1B
M85289-12	15.9	heparan sulfate proteoglycan 2 (perlecan)
AB000220-03	13.5	sema domain, immunoglobulin domain (Ig), short basic domain, secreted, (semaphorin) 3C
U29343-01	11.2	hyaluronan-mediated motility receptor (RHAMM)

NM_005417-02	10.7	v-src sarcoma (Schmidt-Ruppin A-2) viral oncogene homolog (avian)
U16752-04	70.2	stromal cell-derived factor 1
D21337-07	35.2	collagen, type IV, alpha 6
Y00815-03	16.9	protein tyrosine phosphatase, receptor type, F
X04571-05	13.8	epidermal growth factor (beta-urogastrone)
L12002-06	10.9	integrin, alpha 4 (antigen CD49D, alpha 4 subunit of VLA-4 receptor)

Identification (ID) of only the AFAS probes is shown. The format of the ID is: NCBI accession number of sense gene-serial number given to the AFAS probes, starting from the 5' end of the antisense region. Thus, the ID for the corresponding sense gene can be inferred from the AFAS probe ID. The column "Diff." denotes the minimum percentage of the differences in expression intensities between normal tissue, cancer tissue, and those obtained by using sense probes, and AFAS probes. Only the third greatest value among six values for each patient is shown. The first 44 rows show the list of sense-AFAS probe pairs detecting up-regulated expression of the sense transcript and down-regulated expression of the AFAS transcript in cancer tissues. The last five rows show the list of sense-AFAS probe pairs detecting down-regulated expression of the sense gene and up-regulated expression of the AFAS gene in cancer tissues. These two lists are separated by a line.

### The biological relevance of sense-AFAS genes can be inferred from their expression patterns

To obtain a global view of the functions of the genes corresponding to the sense-AFAS transcript pairs showing altered expression balances among normal and cancer cells (shown in Table 1 and Additional file 2: Table S4), we classified the known functions of the sense genes on the basis of the annotations given in the Atlas Human Cancer 1.2 Array (Additional file 2: Table S5). Approximately 30% of the sense genes showing altered expression balances with their corresponding AFAS genes were oncogenes, tumor suppressor genes, or genes related to cell cycle function. This finding demonstrates that the design of our AFAS probes had the capacity to efficiently detect transcripts originating from the antisense strand of genes related to oncogenesis.

**Figure 5:**
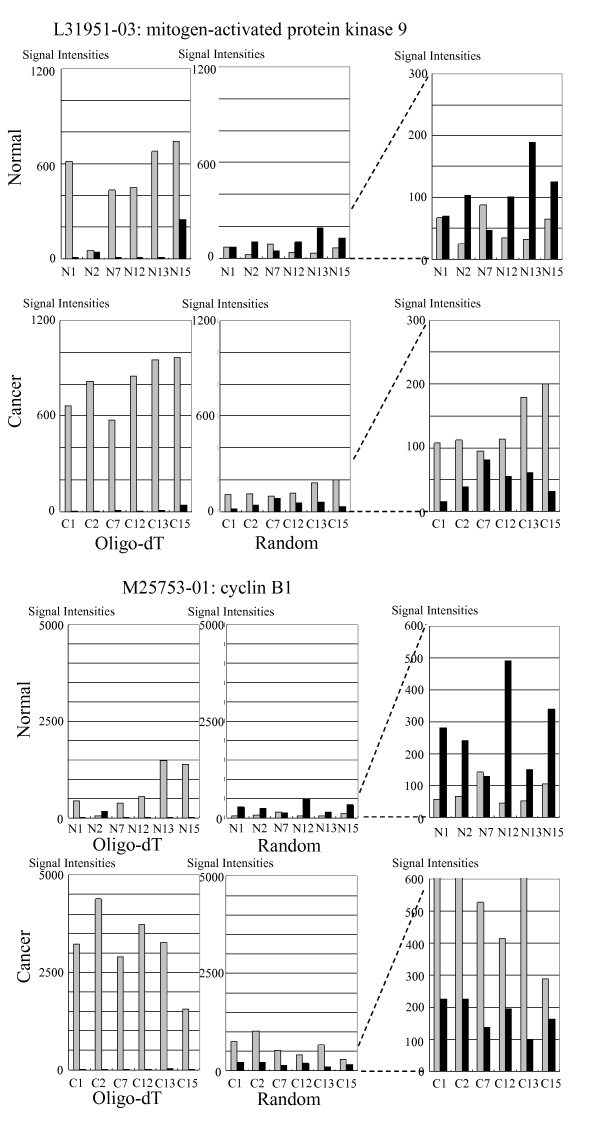
**Two examples of microarray expression levels for sense-AFAS probe pairs where the sense gene is up-regulated and its AFAS gene is down-regulated**.

**Figure 6:**
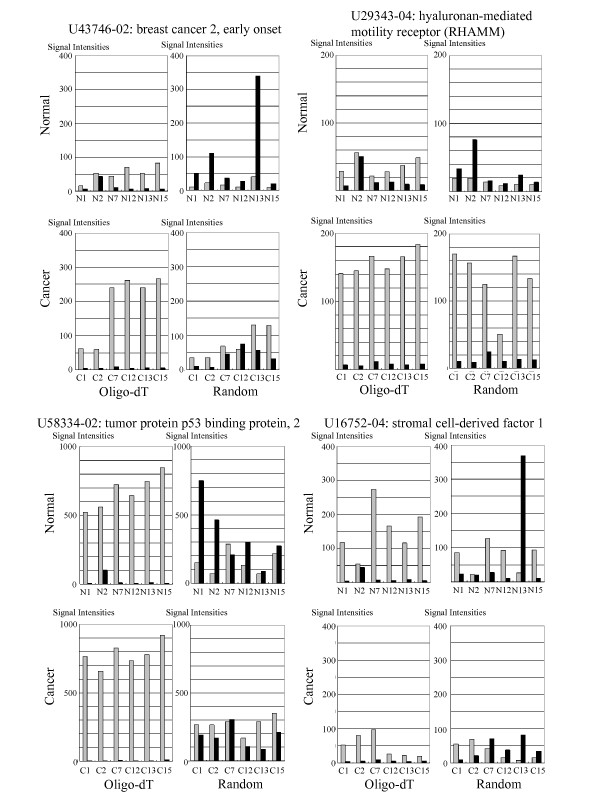
**Examples of microarray expression levels for sense-AFAS probe pairs detecting altered expression balances**. U43746-02, U29343-04 and U58334-02 are examples of cases where the sense gene is up-regulated and its AFAS gene is down-regulated in colon cancer tissues. U16752-04 on the lower right is an example with the opposite tendency where the sense gene is down-regulated and its AFAS gene is up-regulated in colon cancer tissues.

We generated bar graphs for the sense-AFAS pairs shown in Table 1 and displayed the clearest cases in Figures 5 and 6. One of the examples is the sense-AFAS transcript pair detected with the sense probe for mitogen-activated protein kinase 9 (MAPK9) and the corresponding AFAS probe, L31951-03 (Figure 5). In this case, the overall expression level of the sense gene was higher in cancer tissues than in normal tissues, and vice versa for the AFAS gene, based on the generation of target cDNAs with the random priming approach. Four patients (No. 2, 12, 13, and 15) showed clear expression balance alterations of the sense-AFAS transcripts. The oligo-dT priming approach tended to give higher expression levels of the sense genes than did the random priming approach, presumably because of the higher efficiency of oligo-dT priming for labeling transcripts with poly(A) tails. However, the random priming approach was more efficient than the oligo-dT priming approach in allowing the detection of the putative antisense transcripts (AFAS transcripts) as well as the detection of the changes in expression level of these transcripts in normal tissue and cancer tissues. Furthermore, the expression patterns of AFAS transcripts were more likely to be different with the two priming approaches (average correlation coefficient = 0.143) than those of the sense genes (average correlation coefficient = 0.358, *P *= 2.2×10^-16^, Mann-Whitney test). This result suggests that the sets of transcripts originating from the AFAS regions might differ depending on the priming method and that random priming is the more efficient approach for detecting these transcripts.

In some cases, multiple AFAS probes simultaneously detected altered expression balances of specific sense-AFAS transcripts. For example, three AFAS probes designed to detect the transcripts encoded in the antisense strand of BRCA2 (breast cancer 2, early onset, U43746) revealed altered expression balances for the sense-AFAS transcripts originating from this gene (Table 1). This result suggests that for BRCA2, multiple transcriptional units are under the same regulatory control or, alternatively, that a single long transcription unit arises from the antisense strand. On the other hand, the expression levels detected by some AFAS probes were limited to a specific region of a gene. For example, of the five AFAS probes designed to detect the transcripts encoded in the antisense strand of cancer-related gene ADAM metallopeptidase domain 17 (ADAM17, U69611) only U69611-04 revealed an altered expression balance with the sense transcript (Additional file 1: Figure S5). In this case, it is possible that the AFAS probe maps to the transcriptional unit arising from the limited region of the antisense strand. We have preliminary results suggesting that transcripts detected by AFAS probes are more likely to be situated at the 5' end of the corresponding sense genes (Additional file 1: Figure S6), a result that we also reported in our previous study [11].

## Discussion

In the present study we screened for novel antisense transcripts using our previously established microarray platform with AFAS probes and identified those expressed at different levels in cancer tissues and normal tissues (Table 2). Although many studies have focused on investigating the expression of sense genes with poly(A) tails in cancer tissue, very few studies at a genome-wide level have investigated antisense genes with no poly(A) tail. Our previous [7,10,11] and present studies suggest that such antisense genes are transcribed from many of the sense genes and should be included in a thorough investigation of the gene regulatory networks underlying oncogenesis.

**Table 2:** Summary of the sense and AFAS transcripts that showed different levels of expression in cancer tissues and in normal tissues

		AFAS probes		Corresponding sense genes	
All		2376	(100%)	501	(100%)
AFAS Expression level ≥100	in normal tissue (dT)	213	(9%)	153	(31%)
	in cancer tissue (dT)	183	(8%)	138	(28%)
	in normal tissue (Random)	931	(39%)	392	(78%)
	in cancer tissue (Random)	908	(38%)	388	(77%)
AFAS expression change in cancer tissue	Cancer ≥ Normal×2 (Random)	71	(3%)	56	(11%)
	Normal ≥ Cancer×2 (Random)	101	(4%)	87	(17%)
Sense-AFAS pair having altered expression balance	Sense up, AFAS down (Random)	44	(2%)	39	(8%)
	Sense down, AFAS up (Random)	5	(0%)	5	(1%)
	Sense up (dT), AFAS down (Random)	24	(1%)	17	(3%)
	Sense down (dT), AFAS up (Random)	22	(1%)	16	(3%)

Expression levels for "Expression level ≥ 100" and "Expression increased by" were calculated based on median expression levels among patients for each probe.

Hierarchical clustering of expression ratio of sense and antisense genes followed by heat map creation is a powerful approach to characterize genomic regions from the view point of expression balances among the two strands; it shows genomic regions where either sense or antisense strand is highly expressed compared to the other strand. We expected that for most of the genomic regions, sense genes are highly expressed compared to antisense genes especially because sense genes are well-known whereas most of their antisense counterparts are unknown. This was the case for expression data obtained by oligo-dT priming, but not for those obtained by random priming. We found that 12.9% of sense-AFAS probe pairs have higher expression, by more than threefold, of the AFAS probe compared to the sense probe as clearly observed in heat map (Additional file 1: Figure S4). Also some of AFAS probes showed such high expression specifically in either normal or cancer tissues (Figure 4).

Three antisense transcripts detected by our AFAS probes showed a similar pattern of expression by RT-PCR (Figure 3). More complete validations of antisense transcripts detected by our microarray platform were conducted by RT-PCR, Northern analysis, and *in situ *hybridization in our previous work [11]. Our observed expression patterns of sense genes among normal and cancer tissues were consistent with those in previously published work [23].

The reverse transcription step employed to prepare cDNA for microarray analysis was shown to generate antisense artifacts [24]. This could be a potential problem for our random priming approach since we labeled cDNA generated by reverse transcription. If the synthesis of such artifacts was frequent, we would observe a positive correlation between expression levels of sense and antisense transcripts. However, we observed no such correlation in random priming. Therefore, we suggest that the level of artifacts arising from random priming is minimal. We observed many cases that cannot be attributed to artifacts where the expression level of the antisense transcripts is much greater than that of the corresponding sense transcripts (Figures 5 and 6). Although some of these antisense transcripts might be attributed to transcriptional noise [25], which is possibly enhanced in differentiating cancer cells, we expect that the certain number of these antisense transcripts are functional and include non-coding RNAs. The genomic positions of our detected antisense RNAs are biased towards the 5' end of sense genes (Additional file 1: Figure S6 and [11]). This observation is consistent with previous reports [5,26] and transcriptions of some of these antisense RNAs are suggested to be a part of processes to maintain chromatin structures for subsequent gene regulation [27].

There are several biological interpretations for the function of antisense transcripts detected by AFAS probes showing altered expression patterns between normal and cancer tissues. One interpretation is that they are the result of oncogenesis. For example, during the process of de-differentiation in oncogenesis, sense or antisense RNA expression could be altered. Another interpretation is that the altered expression pattern of the antisense transcripts might be attributed to processes leading to cancer. Some antisense transcripts may function in the cell by expressional control of sense genes through epigenetic regulation [21,28]. The recently discovered non-coding RNA *p15AS*, which is encoded by the antisense strand of the tumor suppressor gene *p15*, was shown to regulate the expression of *p15 *through heterochromatin formation [21]. This study revealed that *p15AS *can suppress the expression of *p15 *and thus *p15 *and *p15AS *have altered expression balances among normal and leukemia cells; *p15 *was down-regulated in leukemia cells, whereas *p15AS *was up-regulated [21]. Although identification of antisense RNAs which suppress sense transcripts based solely on microarray data is very difficult, we extracted 49 sense-AFAS probes showing such altered expression balances (Table 1).

We suspect that Table 1 contains a number of sense-AFAS gene pairs with an important role in the regulatory networks underlying oncogenesis. One such example is the sense-AFAS probe pair X07876-03, which detects expression of wingless-type MMTV integration site family member 2 (WNT2). WNT2 is known to be up-regulated in colon cancer and this up-regulation may be an underlying cause of oncogenesis [29,30]. Another example is U58334-02 (Figure 6), which detects expression of tumor protein p53 binding protein 2 (TP53BP2). TP53BP2 stimulates apoptosis by interacting with the tumor suppressor *p53*. Since this process does not contribute to oncogenesis, the altered expression balance displayed detected by U58334-02 may be the result of oncogenesis. For cadherin 3 (CDH3), the expression of which is detected by the sense-AFAS probe pair X63629-03 (Table 1), a previous study showed that its expression is increased in breast cancer [31]. Thus, it is possible that the expression of CDH3 is also increased in colon cancer. Clues to the function of CDH3 may be gained from investigations of the effect of CDH3 up-regulation on oncogenesis.

Sense-antisense gene pairs that are not directly related to cancer might also show altered expression balances in cancer tissues. Besides the 49 sense-AFAS pairs, we identified 68 sense-antisense transcript pairs among those identified [10] in colon cancer patients showing altered expression balances as already described (Figure 1, Additional file 1: Figures S1-2 and Additional file 2: Table S1).

Cell population heterogeneity should be taken into account in microarray gene expression analyses since it might cause the expression level of an antisense transcript may to be altered and be masked by the expression patterns of other cell types. Thus, clear expression changes might be observed by transcriptional analysis at a single-cell level. Further investigations involving knock-down or over-expression experiments would contribute to the identification of antisense transcripts with an important regulatory role.

For some sense genes, multiple AFAS probes showed a high level of gene expression, whereas for the other sense genes, only a single AFAS probe showed a high level of expression. In the former case, long transcripts or multiple short transcripts are transcribed from the antisense strand. Since our AFAS probes are designed for every 500 bases, the resolution is low. Further experiments such as Northern blot analysis could be used to accurately determine the size of these transcripts.

Finally, our microarray approach, combined with biological validation, provides an efficient approach to elucidate the regulatory pathways underlying altered expression of antisense transcripts and might uncover important roles for these transcripts in oncogenesis. As a result, this approach has the potential to lead to the identification of novel biomarkers for cancer.

## Conclusion

This study analyzed the expression patterns of well-known genes and their putative transcriptional units originating from the corresponding antisense strand using our original probes designed specifically to detect antisense strand transcripts. We discovered that many antisense regions are transcribed and that most of these transcripts are likely to be poly(A)-. Furthermore, we identified antisense transcripts with altered expression in cancer tissues as well as those in sense-antisense transcript pairs displaying altered expression balances in normal tissue and cancer tissue. Taken together, these results provide further insight into the regulation of genes and their antisense transcripts in cancer tissues.

## Methods

### Preparation of tissue samples

For microarray analysis, we prepared colon cancer tissue and normal tissue samples that were ~10 cm away from the cancer tissue, from six patients. Clinical data of the patients are presented in Additional file 2: Table S6. Pathological examinations demonstrated that all tumors included in the study were adenocarcinoma. The extracted normal tissue was composed mostly of normal mucosa. The colon cancer and normal tissue samples were obtained during surgical resection of solitary cancerous lesions in the colon. Each tissue sample was homogenized in liquid nitrogen and total RNA was extracted using TRIzol Reagent (Gibco BRL, Carlsbad, California). Transcripts were labeled with fluorescent dyes by using either oligo-dT or random priming.

The study protocol conformed to the ethical guidelines of the Declaration of Helsinki (1975). All patients provided written informed consent for the analysis of the biopsy specimens and the hospital ethics committee approved the study.

### Probe design and microarray analysis of well-known genes and their AFAS

Microarray probes were designed for sense-antisense transcripts identified by genome mapping of cDNA sequences. Using the global mean scaling method, the expression intensities of probes in each array were normalized to those in brain tissue obtained by oligo-dT priming as previously described [10].

The list of well-known genes suitable for cancer research was obtained from the Atlas Human Cancer 1.2 Array (Cat. No. 634529, Clontech). For each gene, we designed probes for every 500 bps to detect expression from the antisense strand. The optimal position of each probe was determined by using the tools provided by Agilent. We excluded well-known genes if the AFAS probe design failed. Some AFAS probes were designed on the exon-intron boundaries of the sense strand and were designated as "truncated" (Additional file 1: Figure S5). These probes were not included in the analysis shown in Figure 2. We designed a total of 2376 AFAS probes covering 501 well-known genes.

Microarray data used in this study have been deposited in GEO and have been assigned series accession number GSE14397 and GSE14398.

To validate our expression data, we investigated whether well-known genes known to be regulated in colon cancer tissues show similar expression profiles in our system. For each gene, we calculated the median expression level among patients for normal and cancer tissues and assessed whether the median expression level increased or decreased in cancer tissues compared with normal tissues. For this analysis, we used microarray data obtained from oligo-dT primed transcripts because the transcripts of most protein-coding have poly(A) tails. We compared our gene expression data with previously reported gene expression analyses of colon cancer tissues [23]. Of the 245 genes that were previously reported as showing altered expression in colon cancer, 28 genes were included in our list of well-known genes. We showed that 11 of the 14 genes previously shown to be up-regulated in colon cancer had a higher level of expression in cancer tissues compared with normal tissues. We also showed that 12 of the 14 genes previously shown to be down-regulated in colon cancer had a lower level of expression in cancer tissues compared with normal tissues. These results show that our data correlate well with the data of previous studies, thus validating our expression data. Our microarray data obtained from random priming of transcripts were similar, although we did not observe the down-regulation of genes previously shown to be down-regulated in colon cancer.

We note that although there are correlations among expression levels detected by multiple AFAS probes for each well-known gene and also among expression levels for multiple patients detected by each probe, we assumed that there were no such correlations in order to roughly estimate the P-values based on the χ^2 ^tests.

### Detection of antisense transcripts by RT-PCR

We performed RT-PCR assays on commercial total RNA: Human Colon Tumor Total RNA (Clontech), Human Colon Total RNA (Clontech). We also prepared total RNA from HUC-Fm (Normal umbilical cord fibroblast, Cell Bank). For RNA isolation and cDNA synthesis, we treated total RNA with RQ1 RNase-free DNase (Promega) to remove contaminating genomic DNA and then further purified the RNA using Trizol reagent (Invitrogen) according to the manufacturer's recommended protocols. RNA concentration and the 260/280 nm ratios were determined with a NanoDrop ND-1000 spectrophotometer (NanoDrop Technologies). Total RNA (1 μg) was reverse transcribed with 50 U of Super ScriptTM III reverse transcriptase (Invitrogen) using 2.5 ng/μl of strand specific primers according to manufacturer's instructions. Forward primers were used to transcribe cDNA from the (-) strand RNA. cDNA samples were diluted 1:5 in DEPC-treated water prior to real-time PCR.

We performed real-time PCR by using the Thermal Cycler Dice^® ^Real Time System (Takara) for quantification of transcripts. Reactions were done in 10 μl volumes containing 200 nM of each primer, 1.0 μl cDNA, and 5 μl SYBER^® ^Premix EX Taq (Takara). We used the following primer sequences. For GAPDH-AS 5'- AGTCCACTGGCGTCTTCACC -3 and 5' - ATGGTTCACACCCATGACGAAC -3'; for Cxcl12-AS 5'- TCCCTCACATGTCAGTACCTTCA - 3 and 5' - GAATCTTCATGTCCAGGATTGG- 3; for BRCA2-AS 5' - GGAAGTCTGTTTCCACACCTGTC -3 and 5' - CAGGTGGAGGTAAAGGCAGTCTA - 3; for TP53BP2-AS 5' - GCTCTTCACAATGCTGTGTGTG - 3 and 5'-AAACCGATTCCCACAACTGAAG -3. Reactions were run using the manufacture's recommended cycling parameters of 95 °C for 10 s and 40 cycles of 95 °C for 5 s and 60 °C for 30 s. The dissociation curve for each sample was then analyzed to verify the specificity of each reaction. The assays were performed in duplicate and the copy number of each RNA was calculated with Thermal Cycler Dice^® ^Real Time System Software (Takara). The data for each sample were normalized to an internal standard (GAPDH).

## Abbreviations

AFAS: Artificial Antisense Sequence;

## Competing interests

The authors declare that they have no competing interests.

## Authors' contributions

RS wrote the manuscript. RS, YO, YO, and KN conducted bioinformatics analyses. KK, SM, and NO prepared the tissue samples, and KK participated in the design of the experiments. KN, HN, NY, and HK developed the viewer. AK, HY, KA, M.T., and HK supported writing the manuscript. KW and HK conducted microarray experiments. HK designed the experiments and organized the project. All authors have read and approved the final manuscript.

## Pre-publication history

The pre-publication history for this paper can be accessed here:

http://www.biomedcentral.com/1755-8794/4/42/prepub

## Supplementary Material

Additional file 1 **Supplemental figures**. **Figure S1**: Clustering analysis of the expression ratio of the sense and antisense pair of transcripts. **Figure S2**: Changes in expression for sense-antisense gene pairs in cancer tissues, compared with surrounding normal tissues. **Figure S3**: Changes in expression for sense-AFAS gene pairs in cancer tissues, compared with surrounding normal tissues. **Figure S4**: Clustering analysis of the expression ratio of well-known genes (sense transcripts) and their putative antisense transcripts. **Figure S5**: Expression intensities detected by probes designed for the sense gene U69611 and its corresponding AFAS transcript. **Figure S6**: Average Expression levels detected by AFAS probes within every 500 bases with respect to the both termini of sense transcripts. Click here for file

Additional file 2 **Supplemental tables**. **Table S1**: List of sense-antisense gene pairs having altered expression balances in cancer tissues compared to surrounding normal tissues. **Table S2**: List of gene categories of sense-antisense transcripts displaying altered expression balances in normal tissues and cancer tissues. **Table S3**: Percentages of probes detecting positive expression according to criteria supplied by Agilent's platform. **Table S4**: List of sense-AFAS pairs detecting altered expression balances in normal tissues and cancer tissues. **Table S5**: Numbers of sense genes belonging to specified functional categories. **Table S6**: Clinical data of patients. Click here for file
