# Supplementary material for: Comprehensive expressional analyses of antisense transcripts in colon cancer tissues using artificial antisense probes
Source: BMC Med Genomics. 2011 May 16;4:42. doi: 10.1186/1755-8794-4-42 (PMC3125192; doi:10.1186/1755-8794-4-42)
Supplement: Additional file 1 — Supplemental figures. Figure S1: Clustering analysis of the expression ratio of the sense and antisense pair of transcripts. Figure S2: Changes in expression for sense-antisense gene pairs in cancer tissues, compared with surrounding normal tissues. Figure S3: Changes in expression for sense-AFAS gene pairs in cancer tissues, compared with surrounding normal tissues. Figure S4: Clustering analysis of the expression ratio of well-known genes (sense transcripts) and their putative antisense transcripts. Figure S5: Expression intensities detected by probes designed for the sense gene U69611 and its corresponding AFAS transcript. Figure S6: Average Expression levels detected by AFAS probes within every 500 bases with respect to the both termini of sense transcripts. [file 1755-8794-4-42-S1.PPT]

## Slide 1
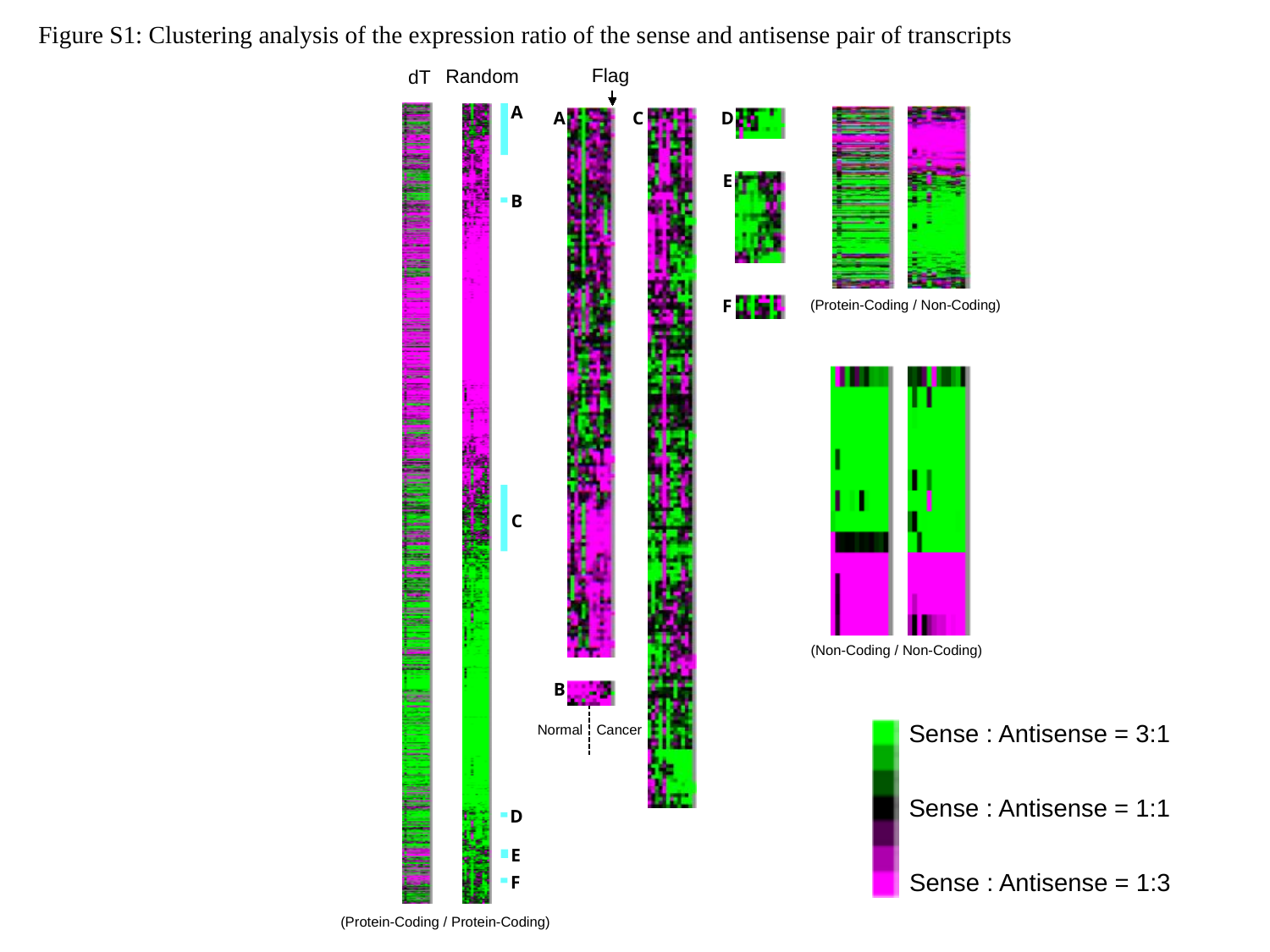

Figure S1: Clustering analysis of the expression ratio of the sense and antisense pair of transcripts
Flag
Random
dT
A
A
C
D
E
B
(Protein-Coding / Non-Coding)
F
C
(Non-Coding / Non-Coding)
B
Sense : Antisense = 3:1
Cancer
Normal
Sense : Antisense = 1:1
D
E
Sense : Antisense = 1:3
F
(Protein-Coding / Protein-Coding)

## Slide 2
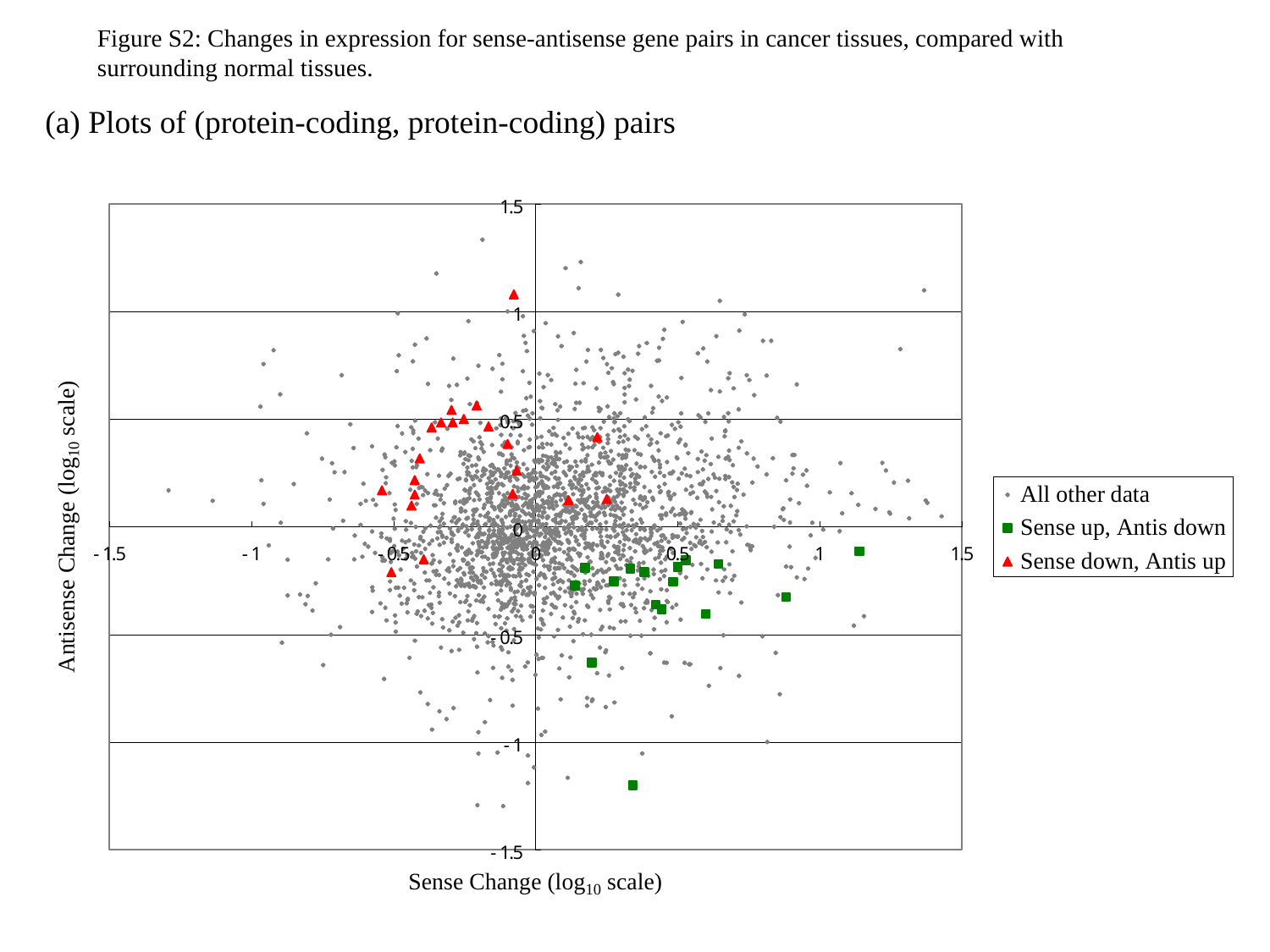

Figure S2: Changes in expression for sense-antisense gene pairs in cancer tissues, compared with surrounding normal tissues.
(a) Plots of (protein-coding, protein-coding) pairs

## Slide 3
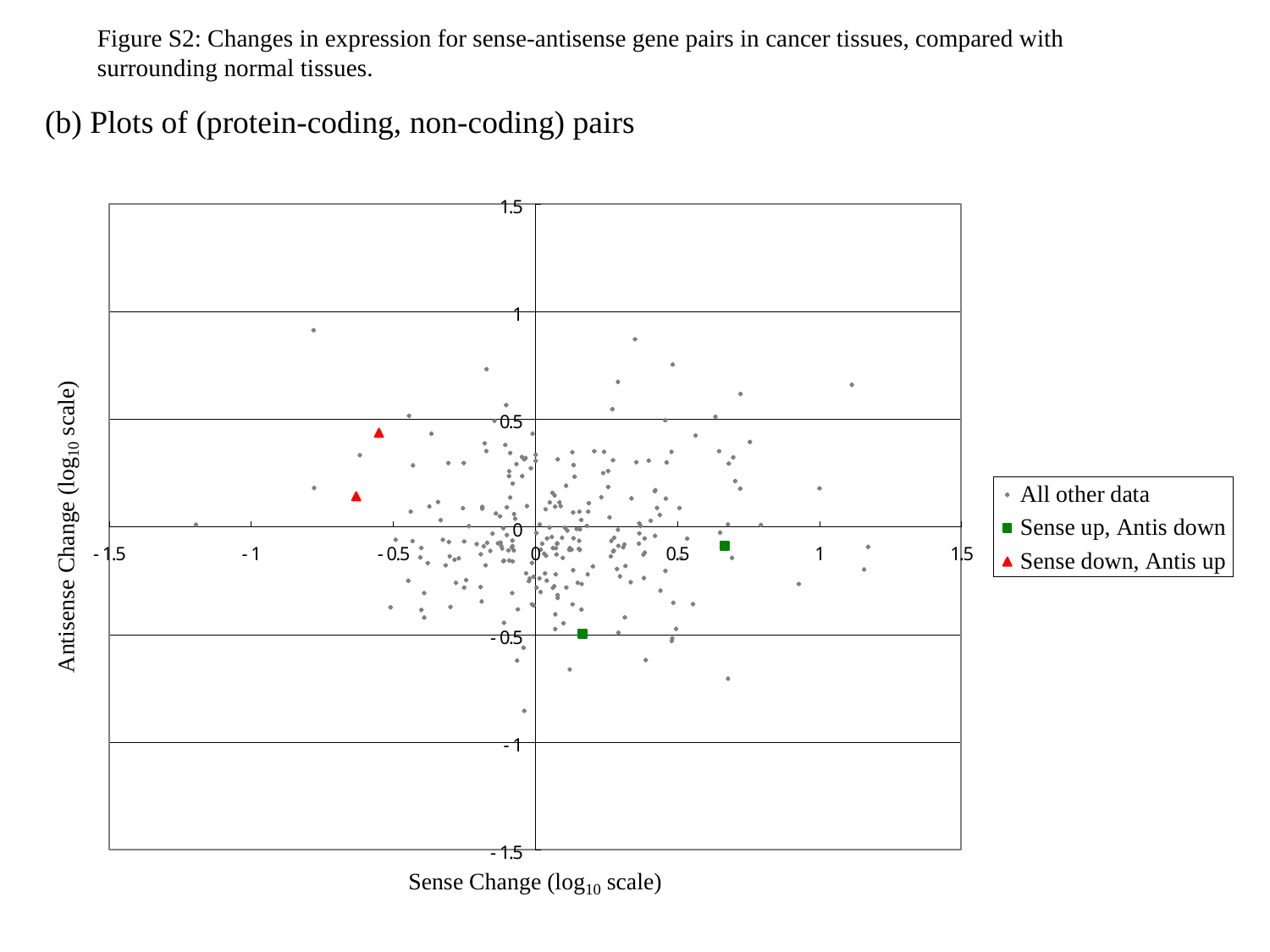

Figure S2: Changes in expression for sense-antisense gene pairs in cancer tissues, compared with surrounding normal tissues.
(b) Plots of (protein-coding, non-coding) pairs

## Slide 4
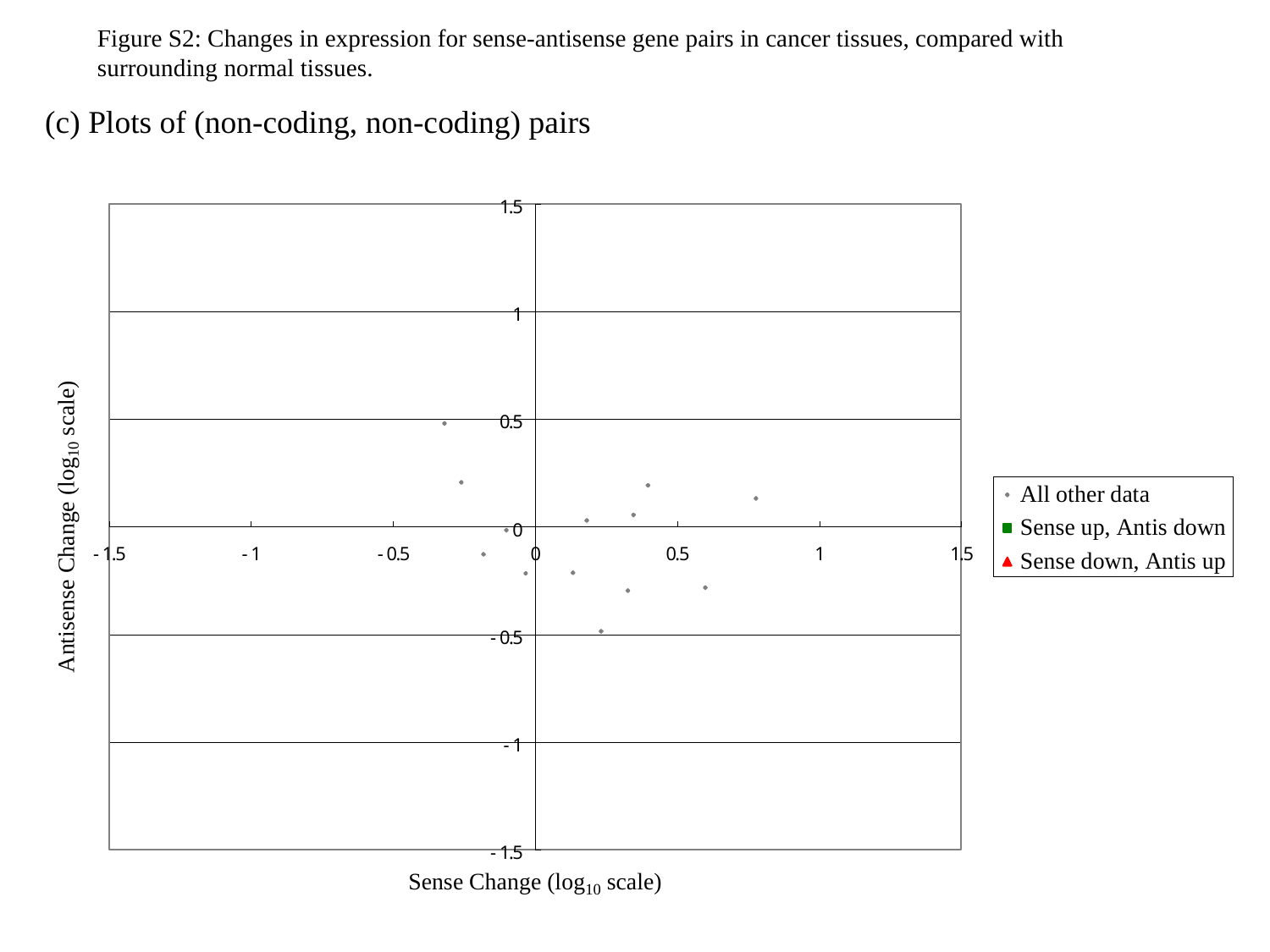

Figure S2: Changes in expression for sense-antisense gene pairs in cancer tissues, compared with surrounding normal tissues.
(c) Plots of (non-coding, non-coding) pairs

## Slide 5
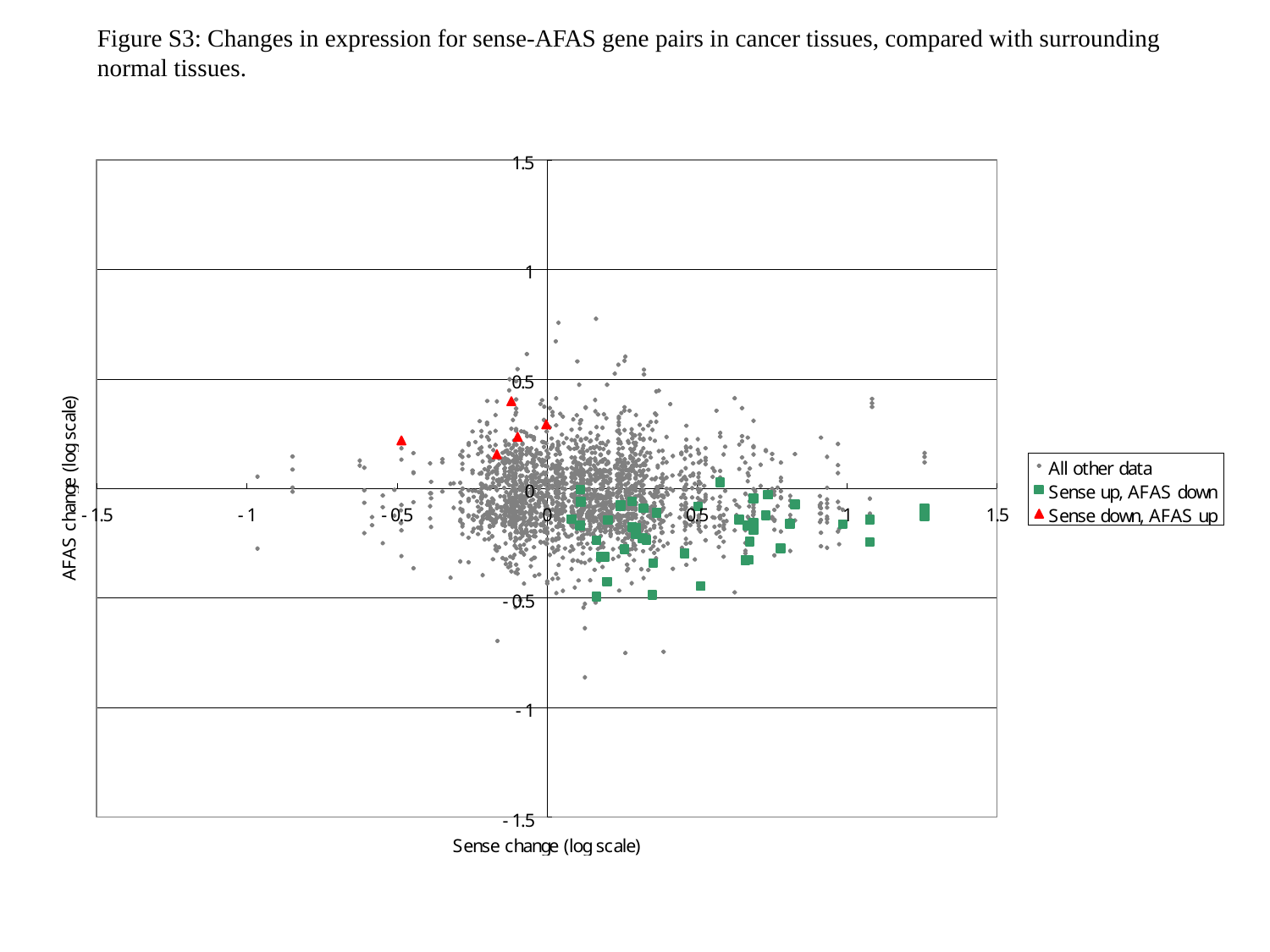

Figure S3: Changes in expression for sense-AFAS gene pairs in cancer tissues, compared with surrounding normal tissues.

## Slide 6
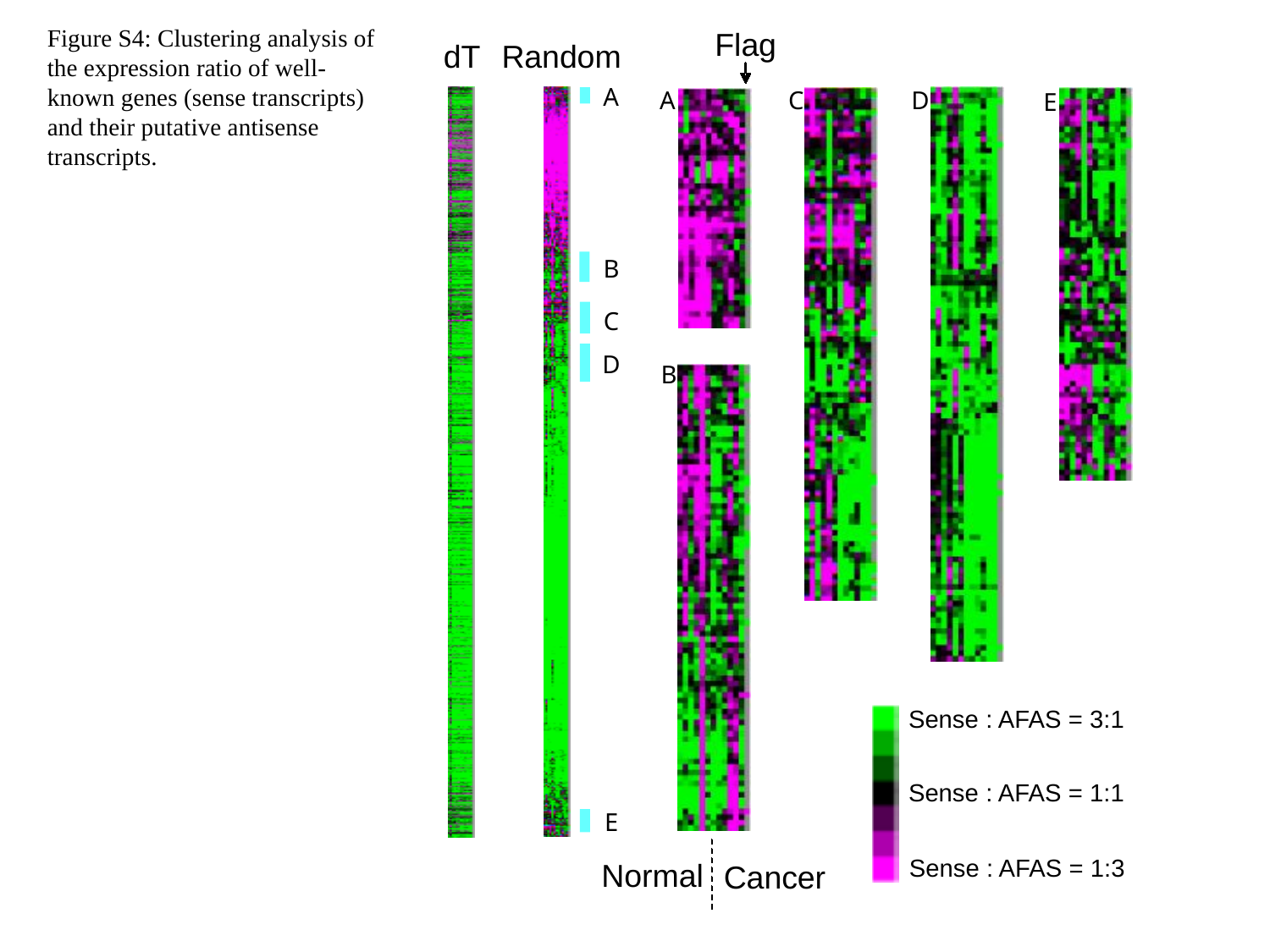

Figure S4: Clustering analysis of the expression ratio of well-known genes (sense transcripts) and their putative antisense transcripts.
Flag
dT
Random
A
A
C
D
E
B
C
D
B
Sense : AFAS = 3:1
Sense : AFAS = 1:1
E
Sense : AFAS = 1:3
Normal
Cancer

## Slide 7
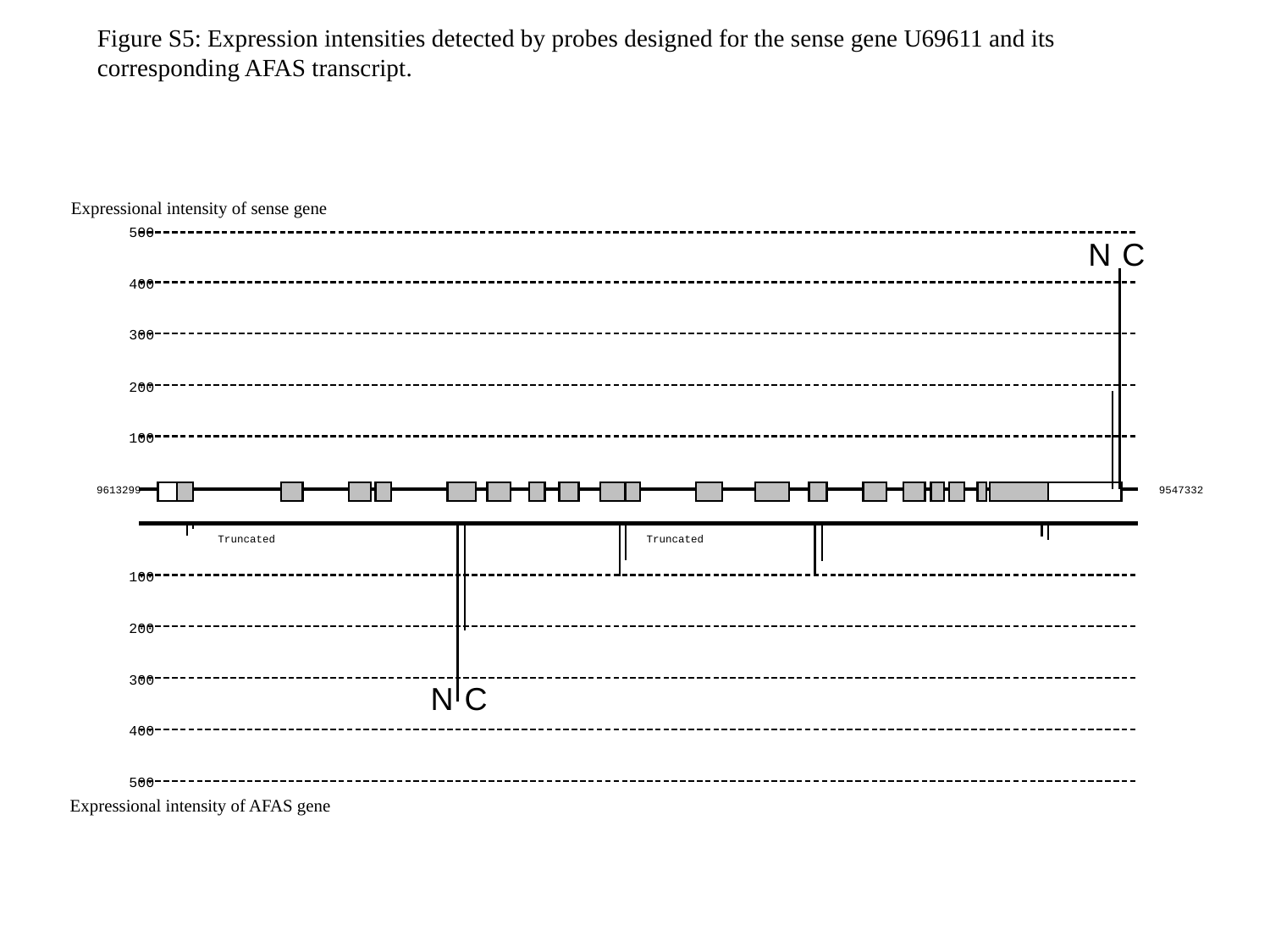

Figure S5: Expression intensities detected by probes designed for the sense gene U69611 and its corresponding AFAS transcript.
Expressional intensity of sense gene
500
C
N
400
300
200
100
9613299
9547332
Truncated
Truncated
100
200
300
C
N
400
500
Expressional intensity of AFAS gene

## Slide 8
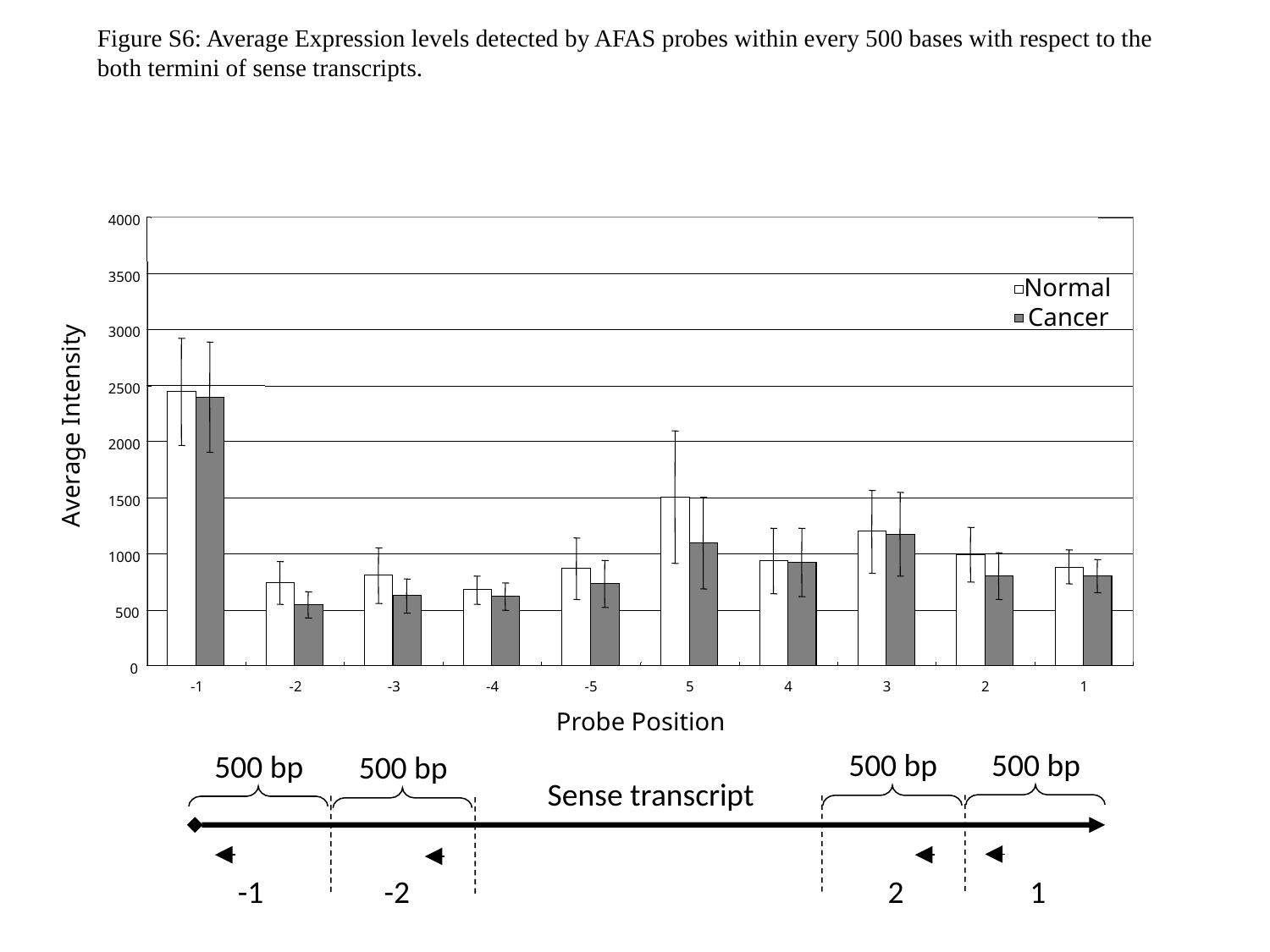

Figure S6: Average Expression levels detected by AFAS probes within every 500 bases with respect to the both termini of sense transcripts.
4000
3500
Normal
Cancer
3000
2500
Average Intensity
2000
1500
1000
500
0
-1
-2
-3
-4
-5
5
4
3
2
1
Probe Position
500 bp
500 bp
500 bp
500 bp
Sense transcript
-1
-2
1
2
